# Automated Detection of Endometrial Polyps from Hysteroscopic Videos Using Deep Learning

**DOI:** 10.3390/diagnostics13081409

**Published:** 2023-04-13

**Authors:** Aihua Zhao, Xin Du, Suzhen Yuan, Wenfeng Shen, Xin Zhu, Wenwen Wang

**Affiliations:** 1 Graduate School of Computer Science and Engineering, The University of Aizu, Aizu-Wakamatsu 965-8580, Japan; 2 Department of Gynecology, Maternal and Child Hospital of Hubei Province, Tongji Medical College, Huazhong University of Science and Technology, Wuhan 430070, China; 3 Department of Obstetrics and Gynecology, Tongji Hospital, Tongji Medical College, Huazhong University of Science and Technology, Wuhan 430030, China; 4 School of Computer Engineering and Science, Shanghai University, Shanghai 200444, China

**Keywords:** endometrial polyps, hysteroscopy, deep learning model, YOLOX

## Abstract

Endometrial polyps are common gynecological lesions. The standard treatment for this condition is hysteroscopic polypectomy. However, this procedure may be accompanied by misdetection of endometrial polyps. To improve the diagnostic accuracy and reduce the risk of misdetection, a deep learning model based on YOLOX is proposed to detect endometrial polyps in real time. Group normalization is employed to improve its performance with large hysteroscopic images. In addition, we propose a video adjacent-frame association algorithm to address the problem of unstable polyp detection. Our proposed model was trained on a dataset of 11,839 images from 323 cases provided by a hospital and was tested on two datasets of 431 cases from two hospitals. The results show that the lesion-based sensitivity of the model reached 100% and 92.0% for the two test sets, compared with 95.83% and 77.33%, respectively, for the original YOLOX model. This demonstrates that the improved model may be used effectively as a diagnostic tool during clinical hysteroscopic procedures to reduce the risk of missing endometrial polyps.

## 1. Introduction

Endometrial polyps are defined as overgrowths of endometrial glands, stroma, and blood vessels from the lining of the uterus. Women of all ages may suffer from this disorder. The peak incidence age is 40–49 years [1,2]. The main symptoms include abnormal uterine bleeding, pelvic pain, infertility, and endometrial polyps, with a malignant transformation rate in the range of 0–13% [3,4].

Hysteroscopic endometrial polypectomy is the standard treatment for patients with symptoms and high-risks [5,6]. However, the complications of this approach—such as intraoperative bleeding, uterine perforation, peripheral organ damage, water intoxication, and intrauterine adhesions—should be of great concern [7]. Water intoxication can induce systemic toxicity and even death, caused by the fluid loading dury the surgery. Experienced hysteroscopists play an important role in reducing these intraoperative and postoperative complications and can make preliminary predictions about the extent of the disease. Therefore, a long period of experience is required for effective hysteroscopy, while junior hysteroscopists may extend a procedure unnecessarily or miss polyps. Therefore, a tool that can assist in diagnosis is urgently needed to fill this gap.

Deep learning has powerful capabilities to learn and recognize patterns in data; therefore, it has been widely implemented in medical image analysis in recent years. Previous studies have been performed to optimize the efficiency of analysis, diagnosis, and treatment strategies using deep learning. Ramamurthy et al. proposed an Effimix model, combining squeeze and excitation layers, along with self-normalization activation layers, with a backbone of EfficientNet B0. Their model achieved a high accuracy of 97.99% in the classification of gastrointestinal diseases [8]. Muruganantham et al. combined ResNet-50 and self-attention mechanisms to localize gastrointestinal lesions in wireless capsule endoscopy images, achieving a classification accuracy of 95.1% and 94.7% on two publicly available datasets—the bleeding dataset and the Kvasir-Capsule dataset, respectively [9]. Object detection algorithms based on deep learning provide the location and classification of lesions in medical images as advantageous tools for aiding in clinical diagnosis. Jha et al. proposed ColonSegNet to detect, localize, and segment polyps in colonoscopy images, with a better trade-off between an average precision of 80.0% and mean IoU of 0.810, and a maximum speed of 180 frames per second for the detection and localization task [10]. More studies have demonstrated the usefulness of object detection algorithms for lesion detection in endoscopic images [11,12,13]. Some researchers have also applied deep learning to the identification of endometrial lesions. Hodneland et al. used a three-dimensional convolutional neural network (CNN) to automate the segmentation of endometrial cancer in magnetic resonance images (MRIs) [14]. They claimed that the CNN-based segmentation accuracy and tumor volume estimation were equivalent to the results achieved by radiologists’ manual segmentation. Kurata et al. segmented uterine endometrial cancer via multi-sequence MRI with a CNN [15]. Zhang et al. used a VGGNet-16 model to classify endometrial lesions from hysteroscopic images [16]. Takahashi et al. proposed a system based on deep learning of hysteroscopy images to localize endometrial cancer lesions automatically [17].

To date, endometrial lesion evaluation based on computer-aided analysis has mainly focused on the object classification or segmentation using computerized tomography images, MRI, and ultrasound images. The application of deep learning is rarely employed in the object detection of endometrial polyps using hysteroscopy. In this study, we investigate the use of deep neural networks for the identification and location of endometrial polyps. This would assist doctors in detecting lesions, lessen their workload, improve the accuracy of diagnosis and treatment and, finally, reduce the risk of endometrial cancer. Our contributions are summarized as follows:

- We proposed group normalization in the deep learning model YOLOX to improve the performance of real-time detection of endometrial polyps from hysteroscopic images.
- A video adjacent-frame association algorithm was applied in the post-processing stage. The algorithm effectively solved the problem of the original YOLOX, i.e., unstable polyp detection.
- We present the first application based on deep learning to detect endometrial polyps from hysteroscopic images.

The rest of this paper includes a Methods section introducing the data sources, methods, and evaluation metrics of this study. The experimental results are presented in the Results section. Discussion of the results and limitations of this study is presented in Discussion section. We end with some conclusions and prospects for future studies in the Conclusions section.

## 2. Methods

### 2.1. Datasets

The training and test datasets were collected from a consecutive series of patients at the Maternal and Child Hospital (MCH) in Hubei Province during 2008–2019 and Tongji Hospital (TJH) at Huazhong University of Science and Technology during 2018–2020.

**Figure 1:**
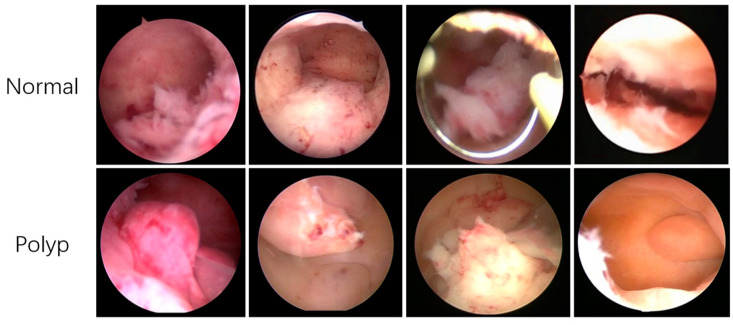
Examples of the hysteroscopic images used in this study.

The training set included videos from 323 cases diagnosed with polyps in the MCH. A total of 7313 and 4526 images with and without polyps, respectively, were extracted from these cases for training. Our test sets comprised cases from two hospitals (MCH and TJH). The MCH test set served as the internal test data and comprised videos from 48 and 183 cases with and without polyps, respectively. For the external test data, the TJH test set comprised videos from 150 and 50 cases with and without polyps, respectively. In addition, we employed five videos with polyps from the TJH to evaluate the real-time detection performance of the proposed model. Figure 1 displays examples of the hysteroscopic images used in this study.

**Figure 2:**
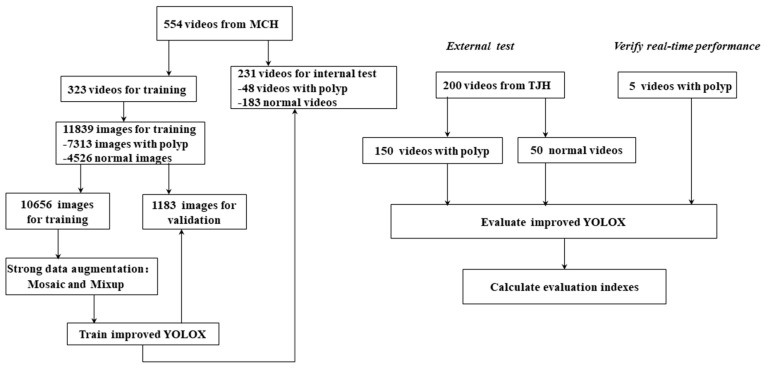
Development and validation flowchart. MCH, Maternal and Child Hospital; TJH, Tongji Hospital.

Figure 2 depicts the general processing flow of the proposed method. Videos from the two test datasets were utilized to evaluate the accuracy, specificity, and sensitivity of the model.

### 2.2. Data Preprocessing

**Figure 3:**
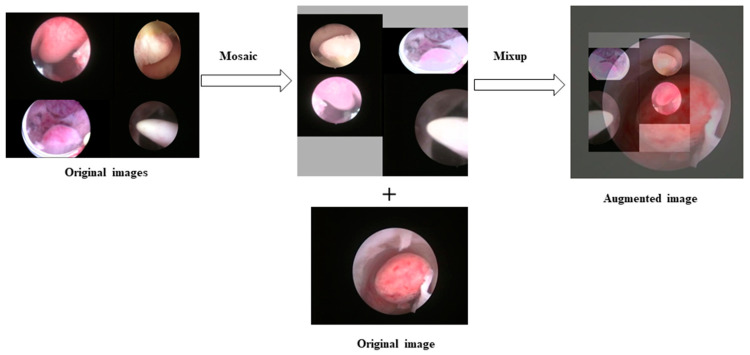
Data augmentation implementation process.

Two gynecologists (W.W. and X.D.) annotated the training set manually, selecting normal images and images containing at least one polyp. To enhance the generalization and robustness of the deep learning model, mosaic and mixup augmentation methods were used in data preprocessing [18]. Mosaic augmentation involves the following steps: (1) randomly select one set of coordinates from the coordinates of the center points of four images to be included in the output image; (2) randomly choose the indices of the other three images and read their corresponding labels; (3) resize each image to 640 × 640 pixels while preserving its aspect ratio; (4) based on the rules of up, down, left, and right, calculate the position where each image should be placed in the output image; (5) crop the output image. Mixup augmentation is implemented as follows: (1) use random jitter augmentation; (2) randomly apply flip augmentation; (3) mix the original image and the processed image using a ratio of 1:1. The data augmentation implementation process is shown in Figure 3.

### 2.3. Improved YOLOX

YOLOX is a real-time object detection model that utilizes convolutional neural networks to detect and classify objects in images and videos [18]. YOLOX has demonstrated excellent performance and processing speed in real-time object detection applications. YOLOX significantly improved YOLO (You Only Look Once) models by introducing an anchor-free design and a decoupled head structure, in addition to stronger data enhancement methods. Through these modifications, YOLOX demonstrates a superior performance in speed and accuracy, making it one of the most advanced detectors currently available. In this study, we propose a model based on YOLOX for hysteroscopic detection. The proposed model may enhance the diagnostic capabilities of doctors during clinical hysteroscopic surgery.

#### 2.3.1. Group Normalization

The original YOLOX used batch normalization (BN) to improve accuracy [19]. However, BN requires a sufficiently large batch size to work effectively, because normalization is performed over the entire batch of input data. A small batch may lead to an inaccurate estimation of batch statistics and, therefore, increase errors. However, increasing the batch size may reduce the training effect and stability. As the batch size increases, more memory is required to store and process the data. If the memory capacity is insufficient, memory errors and crashes may occur. For example, because all hysteroscopy images are resized to 640 × 640 pixels in this study, a large batch size is required for effective training, which may lead to insufficient memory. As a result, batch size and memory capacity should be balanced to achieve optimal training performance. To meet this challenge, a group normalization (GN) method was adopted to use smaller batch sizes without sacrificing performance [20]. The main difference between GN and normal normalization methods lies in using S_i_ at each pixel point, where S_i_ is the set of pixels by which the mean and standard deviation are calculated for normalization. A four-dimensional vector i = (i_N_, i_C_, i_H_, i_W_) is used to index the features in the order of (N, C, H, W), where N represents the batch axis, C the channel axis, and H and W the spatial axes of height and width, respectively. When using GN, the value of S_i_ is defined as follows:

(1) $$S_{i}=\{k|k_{N}=i_{N},\left\lfloor \frac{k_{C}}{C/G}\right\rfloor =\left\lfloor \frac{i_{C}}{C/G}\right\rfloor \}$$

where G is the number of groups (set to 32 by default), *C*/*G* is the number of channels per group, “$k_{N}=i_{N}$” refers to pixels within the same batch, and “$\left\lfloor \frac{k_{C}}{C/G}=\frac{i_{C}}{C/G}\right\rfloor$” refers to pixels with the same channel index and within the same group.

The GN method divides the channels into groups and calculates the mean and variance of each group for normalization. The main advantage of GN is that it normalizes the activations within each group rather than over batch sizes. Using GN, effective training can be achieved with relatively smaller batch sizes to improve the efficiency and effectiveness of YOLOX.

**Figure 4:**
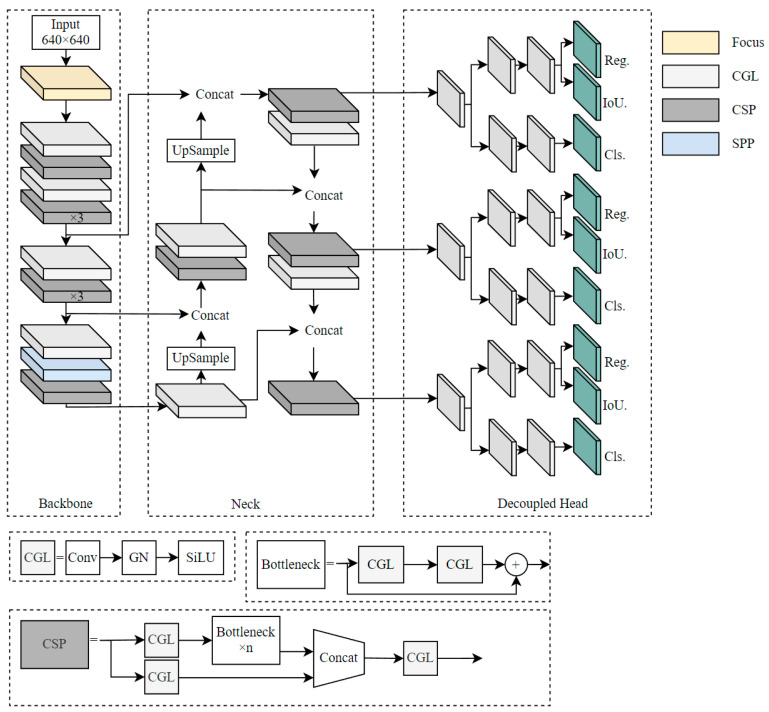
The network structure of the improved YOLOX. Modules are differentiated by color, as shown in the legend. The training images are resized to 640 × 640 pixels as the inputs of the model. The symbol “×3” means that there are three bottleneck modules.

**Figure A1:**
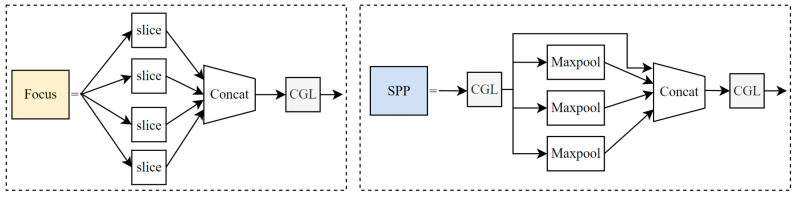
The detailed structure of the focus and SPP modules.

The network structure of the improved YOLOX is shown in Figure 4. The model network architecture consists of three main components: the backbone, neck, and decoupled head. The backbone network is responsible for extracting image features and comprises cross-stage partial connections (CSP), spatially separable convolution (SSP), bottleneck modules, and focus modules [18]. The CGL module is the smallest component in the network architecture of YOLOX. It is composed of convolution operations, GN, and SiLU activation functions. The detailed structure of the SPP and focus modules is shown in Figure A1 in the Appendix A. CSP modules enhance feature complexity and diversity by sharing information across multiple layers through cross-stage local connections. SSP modules employ spatially separable convolutions to reduce model parameters and improve computational efficiency. Bottleneck modules increase feature expressiveness and reduce computational costs by employing dimensionality reduction and expansion techniques. The focus module—a specialized convolution module—decomposes input feature maps, thereby reducing convolutional computation while improving the detector’s sensitivity to small objects.

The neck component is designed to generate a top-down pathway, which starts with the deepest feature map from the backbone network and progressively upsamples the feature map to produce higher-resolution feature maps. Simultaneously, a lateral connection pathway merges the upsampled features with corresponding features from the bottom-up pathway at each level. This process ensures that the multiscale feature maps retain high-level semantic information while incorporating finer details from lower-level features. Multiscale feature maps are utilized to further enhance the model’s performance.

The decoupled head module serves as the core of the detector, responsible for classification and regression of features. This module adopts a decoupled head and anchor-free design, which not only reduces the number of parameters and increases the detection speed but also improves the detection accuracy. Moreover, an advanced label-assignment strategy is employed to address label imbalance and reduce the detector’s propensity for errors, thereby elevating the model’s performance.

#### 2.3.2. VAFA Algorithm

In the post-processing stage, we used a perceptual hash algorithm (PHA) to address the issue of unstable object detection boxes. The PHA calculates the similarity of adjacent frames and uses this information to stabilize the detection boxes in similar frames. This helps to improve the accuracy and consistency of our object detection results. The PHA uses a feature vector called a “fingerprint” to represent an image. It then compares the fingerprints of different images to determine their similarity. The smaller the difference between the fingerprints, the more similar the images. After extensive testing, we found, through trial and error, that when the Hamming distance between two images was ≥9, the images would no longer be considered similar. Using this threshold enabled us to identify and fix unstable detection boxes in our object detection results. This algorithm is defined as a video adjacent-frame association (VAFA) algorithm. Its implementation is as follows:

1. When five or more adjacent frames are detected as containing a “polyp”, the similarity between the current frame and the next frame is calculated.
2. If the similarity is <9, the object detection box of the current frame is assigned to the next frame, until the similarity between frames is no longer <9.

**Figure 5:**
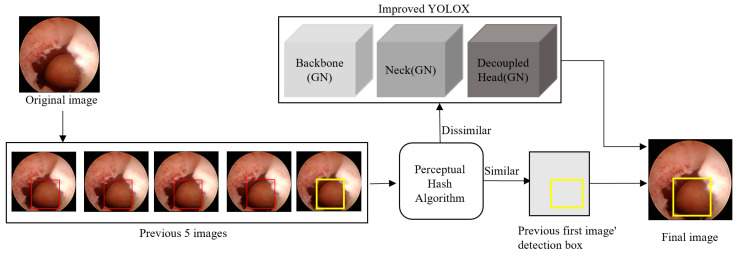
Overview of our improved YOLOX method. GN is applied in the backbone block, neck block, and decoupled head block of the original YOLOX model. The perceptual hash algorithm (PHA) is utilized to compute the similarity between adjacent frames. GN, group normalization.

This process helps to stabilize the detection boxes and improve the accuracy of our object detection results, as shown in Figure 5. Video S1 shows the performance of our proposed model with VAFA algorithm in detecting endometrial polyps.

### 2.4. Model Training

The loss function *L* for the proposed model comprises three components: bounding box regression loss $L_{reg}$, classification loss $L_{cls}$, and object loss $L_{obj}$.

(2) $$L={reg}_{weight}*\frac{1}{N_{pos}}L_{reg}+\frac{1}{N_{pos}}L_{cls}+\frac{1}{N_{pos}}L_{obj}$$

where $N_{pos}$ is the number of positive labels in each training batch, and ${reg}_{weight}$ is a balancing coefficient. The classification loss is used to classify an object to one of the predefined classes, and the bounding box regression loss refines the bounding box around an object to make its detected location more accurate. The object loss *L* predicts the probability of an object being present in an image. Its three components are combined to optimize the model and improve its performance in detecting objects in images. To enhance the regression loss with the other components of the loss function in the YOLOX model, ${reg}_{weight}$ was set to 5.0 by trial and error. This ensures that the model can refine the bounding boxes around detected objects accurately and improve its performance in object detection.

During the training phase, the MCH training data were randomly divided into training and validation sets using a 9:1 ratio. YOLOX-S was used as a baseline model for comparing performance. The input size for the neural network was 640 × 640 pixels, the input images were augmented automatically via the methods introduced in Section 2.2, and augmentation was turned off for the final 15 epochs. We specified the iteration rate as one per 10 epochs except for the last 15 epochs, when validating the validation set and calculating the mean average precision (mAP). In addition, validation was performed after each iteration of the last 15 epochs. The mAP was calculated by comparing a ground-truth bounding box with the detected box. The higher the mAP score, the more accurate the detection result. The weight values of the proposed model were saved, with the highest mAP value as the best checkpoint.

**Figure A2:**
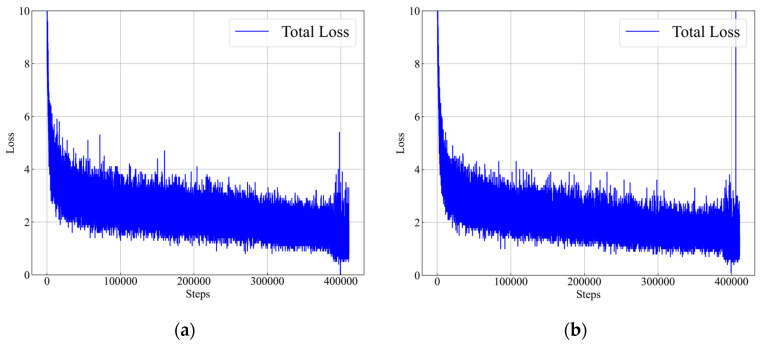
Stepwise loss curves. (**a**) The loss curve of the improved YOLOX model. (**b**) The loss curve of original YOLOX model.

The models were developed using a Python 3.9 and PyTorch 1.12.1 framework and trained on a workstation equipped with an AMD Ryzen 7 3700X 8-Core processor CPU, 32.0 GB RAM, and a NVIDIA GeForce RTX 3060. The training batch size was set to eight, the number of epochs was 300, and half-precision training was enabled. These training hyperparameters remained unchanged during training and were kept constant when training YOLOX models with GN and the VAFA algorithm. Figure A2 illustrates the step-wise loss for both the original YOLOX model and the improved model.

### 2.5. Evaluation

We employed sensitivity, specificity, accuracy, precision, and *F*_1_ to evaluate the performance of the proposed model.

(3) Sensitivity=TP/(TP+FN)

(4) Specificity=TN/(TN+FP)

(5) Accuracy=(TP+TN)/(TP+TN+FN+FP)

(6) Precision=TP(TP+FP)

(7) $$F_{1}-score=2\times Sensitivity\times Specificity/(Sensitivity+Specificity)$$

We evaluated the model at both video and image levels. To calculate the above-mentioned evaluation metrics at the image level, the videos were converted to frames. If the bounding box output by the model overlapped with the location of the polyp in a frame and no bounding box appeared in non-polyp areas, the frame was recorded as a true positive (*TP*). If a polyp was detected in a wrong position, it was counted as a false positive (*FP*). If a frame did not actually contain a polyp and was detected as not hav-ing any polyps, it was recorded as a true negative (*TN*). Otherwise, it was considered a false negative (*FN*). At the video level, if the number of correctly detected frames (i.e., *TP* or *TN*) exceeded half of the total number of frames in the video, the video was considered *TP* or *TN*. Otherwise, the video was considered *FP* or *FN*.

## 3. Results

To assess the efficacy of the modifications to the YOLOX model, we applied it in ablation experiments and calculated the evaluation metrics described in Section 2.5. The models were tested using the checkpoint with the highest mAP. The original model and the model using GN achieved the best checkpoint values after 286 and 260 epochs, respectively. The model using both GN and the VAFA algorithm reached the best checkpoint after 260 epochs, given that VAFA is a post-processing step and does not affect the training process of the model. The results indicated that the improved YOLOX model was able to fit the training data more efficiently, requiring fewer epochs and less time in training, as compared with the original model. This suggests that the modifications were effective in reducing the training time.

To validate the efficacy of GN and VAFA, we conducted ablation studies. The performance of the four models, as shown in Table 1, was evaluated with the MCH and TJH test sets, with a confidence level of 0.4. The images were resized to 640 × 640 pixels for the evaluation. Both the image-level and video-level performances of the models were assessed. Each case in the test set was represented by only a single video.

Table 1 shows that the (per-lesion) sensitivity, specificity, accuracy, precision, and *F*_1_ for the YOLOX+GN+VAFA algorithm with the MCH test set were 100%, 88.52%, 90.91%, 69.57%, and 93.91%, respectively. With the TJH test set, the equivalent results for the YOLOX+GN+VAFA algorithm were 92.0%, 76.0%, 88.0%, 92.0%, and 83.24%, respectively. As shown in Table 2, the YOLOX + GN + VAFA algorithm had a per-image sensitivity of 98.73%, compared with the original YOLOX model’s 96.01%, for the MCH test set. The original YOLOX model’s per-image sensitivity with the TJH test set was 82.07%, whereas that for the YOLOX+GN+VAFA algorithm was 92.92%.

The proposed model is therefore capable of real-time endometrial polyp detection, with a video processing speed of 63 FPS using a NVIDIA GeForce RTX 3060 GPU. This makes it a suitable solution for practical medical applications.

## 4. Discussion

In this study, YOLOX was enhanced for application to the detection of endometrial polyps in hysteroscopic videos.

### 4.1. Evaluation

It was found that the proposed model could achieve high sensitivity and real-time performance. Compared with the original YOLOX model, the proposed model had improved per-lesion sensitivities using both the internal test set from the MCH and the external test set from the TJH, with 100% and 92.0%, respectively. In particular, the per-lesion sensitivity of 92.0% with the external test dataset was significantly superior to the original model’s per-lesion sensitivity of 77.33%, demonstrating the superior generalization capability of the proposed model. This can be attributed to the utilization of the GN method instead of the BN method. BN is strongly dependent on batch size, causing severe variations in the parameter updates and a noticeable decrease in recognition accuracy when small batch sizes are used for training neural networks. Conversely, GN is independent of batch size, leading to consistent performance even with small batch sizes. The integration of the VAFA algorithm further enhances the sensitivity of the proposed model.

In addition, we compared our proposed method with the highly efficient EfficientDet model [21], and the comparison results are shown in Table A1. Table A1 demonstrates that our proposed model yields superior performance to EfficientDet. Specifically, on the MCH test set, our proposed model achieved video-level sensitivity of 100% and image-level sensitivity of 98.73%, outperforming EfficientDet’s corresponding sensitivity of 52.08% and 83.16%, respectively. On the TJH test set, our proposed model also outperformed EfficientDet in terms of sensitivity, achieving video-level sensitivity of 92.0% and image-level sensitivity of 92.92%, compared to EfficientDet’s corresponding sensitivity of 50.0% and 79.23%, respectively. Our proposed model also achieved high accuracy and F1-score values compared to EfficientDet. The details are also listed in Table A1.

As shown in Table 1 and Table 2, the accuracy of the proposed model was worse than that of the original model on the internal test set from the MCH, but similar or even better on the external test set from the TJH. We hypothesize that this issue occurred because all models were trained only with data provided by the MCH, whereas the test set comprised data from both hospitals. Considering variations in the data collection equipment and procedures employed by the two hospitals, there could be an inconsistent distribution between the source and target data. In future work, we will adopt domain generalization methods to address this issue.

The current use of deep learning in hysteroscopic images is mainly focused on the classification of endometrial cancer, with a few studies on endometrial fibroids [16,17]. For example, Török et al. used a fully convolutional CNN to identify the plane between myoma and the normal myometrium, achieving a pixel-wise segmentation accuracy of 86.19% after training the network on 13 cases of video data for 140 epochs [22]. Deep learning research on uterine lesions has mainly focused on MRIs and ultrasound images. Zhang et al. trained and evaluated the LeNet-5 neural network using MRIs from 158 patients with endometrial cancer, achieving an area-under-the-curve value of 0.897 [23]. Dong et al. applied a U-Net neural network to MRI scans, seeking the depth of endometrial cancer invasion and achieving a model accuracy of 79.2%, which was not significantly different from the diagnostic accuracy achieved by radiologists [24]. Xia et al. used a DPA-UNet neural network to integrate hysteroscopy and ultrasonography for the detection of endometrial cancer [25]. Wang et al. achieved automatic endometrial segmentation and thickness measurements for ultrasound images using a 3D U-Net network [26]. Dilna et al. used the MBF-CDNN method to detect uterine fibroids in ultrasound images [27]. Several studies have investigated MRIs of endometrial fibroids using deep-learning-based methods [28,29,30,31]. Overall, these studies demonstrate the potential of deep learning for improving the detection of uterine lesions. The improved YOLOX model developed in this study has shown high sensitivity in the detection of endometrial polyps in hysteroscopic images, and it may be a useful tool for assisting doctors in clinical diagnosis.

To the best of our knowledge, this study is the first to use a deep-learning-based method to detect endometrial polyps based on hysteroscopic images. An improved YOLOX model was used to achieve real-time detection and high accuracy, making it suitable for use in clinical hysteroscopic surgery. In addition, our VAFA algorithm was proposed for the post-processing stage, with the aim of making the object detection box more stable and reducing discomfort for doctors. Our improved YOLOX model was able to achieve its best performance in fewer iterations and with less time overhead compared with the original YOLOX model. Table 1 and Table 2 show that the proposed model demonstrated significantly higher sensitivity than the original model, particularly with the external test set from the TJH. This is advantageous, because the proposed model minimizes the rate of missed detections by doctors. Although the proposed model has lower specificity than the original model, it remains acceptable. Overall, these results demonstrate the potential of deep learning for improving the detection of endometrial polyps in hysteroscopic images.

### 4.2. Limitations

The limitations of this study can be listed as follows:

1. The model showed poor performance in detecting hysteroscopic images with polyps partially occluded by the endometrium.
2. A large floating endometrium can be misdiagnosed as a polyp. We expect that problems 1 and 2 can be addressed by increasing the number of occluded polyp images and background images in the training set.
3. Deep-learning-based object tracking algorithms, such as Deep SORT, have been employed to address the problem of unsteady detection boxes [32]. However, their performance should be improved further. The proposed VAFA algorithm should also be updated, because the object detection display is insufficiently smooth. Therefore, further research is needed to develop more advanced algorithms that would improve the polyp detection performance.
4. A prospective study should be conducted to check that the proposed method performs as expected in real clinical practice.

## 5. Conclusions

In this study, we improved the YOLOX model to achieve higher sensitivity in the detection of endometrial polyps. The VAFA algorithm was also proposed in a post-processing stage to improve the stability of the detection process and enhance its convenience of use by hysteroscopists. The improved model and method showed significant generalizability and stable capacity and could achieve high sensitivity in the detection of endometrial polyps.

Future works will mainly be based on the practical application of this study. Firstly, we expect to explore the feasibility of integrating our algorithm into existing clinical hysteroscopy systems to improve the efficiency and accuracy of endometrial polyp detection. Secondly, we want to optimize the proposed model by combining hysteroscopic videos and medical information for use on mobile devices or web-based platforms. This would facilitate the usage of the proposed method in remote healthcare services or telemedicine systems.

Overall, the improved model may be useful in reducing the missed diagnosis rate in clinical hysteroscopic surgery and improving the detection sensitivity of endometrial polyps.

## Figures and Tables

**Table 1:** Evaluation results of ablation experiments using the MCH and TJH test sets.

Model	Sensitivity (%)	Specificity (%)	Accuracy (%)	Precision (%)	F_1_ (%)
MCH test set					
YOLOX	95.83	95.08	95.24	80.70	95.46
YOLOX + GN	100	89.62	91.77	71.64	94.52
YOLOX + VAFA	100	86.89	88.74	65.71	92.99
YOLOX + GN + VAFA	100	88.52	90.91	69.57	93.91
TJH test set					
YOLOX	77.33	96.0	82.0	98.31	85.66
YOLOX + GN	90.67	80.0	88.0	93.15	85.0
YOLOX + VAFA	91.33	76.0	87.5	91.95	82.96
YOLOX + GN + VAFA	92.0	76.0	88.0	92.0	83.24

**Table 2:** Evaluation results of ablation experiments at the image level using the MCH and TJH test sets.

Model	Sensitivity (%)	Specificity (%)	Accuracy (%)	Precision (%)	F_1_ (%)
MCH test set					
YOLOX	96.01	92.23	92.70	61.39	94.11
YOLOX + GN	98.02	85.54	86.96	46.45	91.36
YOLOX + VAFA	98.68	84.32	85.89	43.69	90.94
YOLOX + GN + VAFA	98.73	84.32	85.95	44.61	90.96
TJH test set					
YOLOX	82.07	91.38	85.66	93.81	86.47
YOLOX + GN	89.25	72.69	82.86	83.88	80.12
YOLOX + VAFA	92.91	70.73	84.40	83.59	80.32
YOLOX + GN + VAFA	92.92	70.41	84.24	83.34	80.11

## Data Availability

The data used in this study are unavailable due to the rules of ethical approvals.

## Supplementary Materials

The following supporting information can be downloaded at: https://www.mdpi.com/article/10.3390/diagnostics13081409/s1, Video S1: A sample of our proposed model with the VAFA algorithm in detecting endometrial polyps.

## Appendix B. Comparison with EfficientDet

Tan et al. proposed an efficient object detection model, EfficientDet, which utilizes a novel model scaling method that uniformly scales the resolution, depth, and width of all of the backbone, feature, and prediction networks simultaneously [21]. Additionally, it incorporates a weighted bidirectional feature pyramid network, which facilitates fast and convenient multiscale feature fusion.

The training environment and parameter settings of EfficientDet are consistent with those of the YOLOX models. If Table A1 shows the comparison between our proposed model and the EfficientDet model.

**Table A1:** Comparison between our proposed model and the EfficientDet model at the image and video levels.

Model (Video-Level)	Sensitivity (%)	Specificity (%)	Accuracy (%)	Precision (%)	F_1_ (%)
MCH test set					
EfficientDet	52.08	97.81	88.31	86.21	67.97
YOLOX + GN + VAFA	100	88.52	90.91	69.57	93.91
TJH test set					
EfficientDet	50.0	98.36	88.31	88.89	66.30
YOLOX + GN + VAFA	92.0	76.0	88.0	92.0	83.24
**Model (Image-Level)**	**Sensitivity (%)**	**Specificity (%)**	**Accuracy (%)**	**Precision (%)**	**F_1_ (%)**
MCH test set					
EfficientDet	83.16	89.34	88.82	43.51	86.14
YOLOX + GN + VAFA	98.73	84.32	85.95	44.61	90.96
TJH test set					
EfficientDet	79.23	89.93	88.97	44.63	84.24
YOLOX + GN + VAFA	92.92	70.41	84.24	83.34	80.11
